# More Convergence on Coercion: Reflecting on Vaccine Mandates in 2026; A Response to Recent Commentaries

**DOI:** 10.34172/ijhpm.9991

**Published:** 2026-05-17

**Authors:** Katie Attwell, Adam Hannah

**Affiliations:** ^1^VaxPol Lab, School of Social Sciences, The University of Western Australia, Perth, WA, Australia.; ^2^Wesfarmers Centre of Vaccines and Infectious Diseases, Telethon Kids Institute, Perth, WA, Australia.; ^3^School of Political Science and International Studies, University of Queensland, Brisbane, QLD, Australia.

## Introduction

 As we finalised our analysis of the adoption of mandatory policies for childhood vaccines in four high-income jurisdictions for our publication, “Convergence on Coercion,”^1^ every country on earth was grappling with how to drive uptake of COVID-19 vaccines.

 The pandemic experience underscored our original analysis: governments adopt coercive vaccination policies in response to both functional and political pressures. Even where countries, particularly in the Global South, faced problems with procurement, access, and service delivery, there was still an urgent need to work out how to get needles into arms as quickly as possible. These pressures were frequently intertwined in the pandemic setting.

 At a functional level, vaccination was the only tool available that could minimise catastrophic deaths and economic disruption. From a political perspective, governments were navigating judgements about their crisis responses, and many were desperate to pivot from the disruptive prevention measures, such as lockdowns, that were depriving people of their freedoms. By comparison, while undeniably coercive, vaccine mandates were often looked upon as an efficient and effective way to shape the behaviour of those who may not have been enthusiastic about receiving a vaccination and thus roll back other social and economic restrictions. Accordingly, we witnessed another “convergence on coercion” for immunisation policy across both hemispheres and a remarkable variety of political and health systems, with a great many governments opting to use some form of coercive policy to drive vaccine uptake.^2^ These coercive measures are now concluded, and many parts of the world have moved on to renewed debates about how to encourage (or require) childhood vaccination in the face of waning uptake. It is an opportune time to respond to the four excellent commentaries on our article published in 2022/2023 and to reflect on the current state of knowledge regarding the politics and policy of vaccine mandates. We do so by orienting the present state of the research field and suggesting some further projects to establish what convergence on coercion in immunisation policy means for the future health and well-being of our interconnected global community.

## Policy Instruments and Policy Mixes Matter

 In their commentary, Odone et al^3^ raise the important point that coercive COVID-19 vaccination measures varied widely in terms of their design, implementation, and relationship with other measures supporting vaccine uptake. Given that governments seem to reach for coercive measures when facing pressure to increase vaccination rates, this highlights the need to better understand the role and impact of coercion in the broader policy “mix” of tools to encourage and enable vaccination, and the potential political and other trade-offs that these design issues pose.

 A notable body of pandemic-focused work centred on the digital instruments used to verify proof of COVID-19 vaccination, often called vaccine passports or vaccine certificates.^4-6^ These instruments often had their genesis as mechanisms for enabling mobility between countries, particularly in the European Union. However, governments were swift to utilise their potential for governing domestic access to employment, economic, and social life, and for the related but distinct purpose of driving up vaccine coverage.^7^ A comparative interpretive policy analysis of how 9 European countries mobilised these instruments for domestic purposes found that they initially included opt-outs for negative tests, so did not function as strict vaccine mandates. However, over time, requirements shifted to strict vaccine mandates in some places, on the back of government discourses that overplayed the policies’ likely efficacy in preventing the spread of disease.^8^ Keeping alternative prevention mechanisms in the mix (ie, negative test opt-outs) helped to reduce the controversy of vaccine passports,^8,9^ but may have also limited the efficacy of these policies in increasing vaccine uptake.

 Beyond the relatively well-studied phenomenon of vaccine passport implementation, also done at a single-country level,^10,11^ the sheer scale and complexity of research required to learn from the wider experience of mandating COVID-19 vaccines presents an almost insurmountable challenge. To draw useful large-scale comparative lessons, we need to know more about what went on in a given country than is possible to glean from the otherwise excellent policy repositories built at the time.^2^ In particular, the question of implementation is usually missing from large-scale datasets on vaccination policies. There is often a considerable variation between what is stated in policy documents or public announcements and what happens on the ground.

 Useful large-scale syntheses would also require attention to how COVID-19 policies connected to pre-existing childhood vaccination policies, including these policies’ ongoing effectiveness in driving uptake during and after the pandemic. However, again, we can rarely know enough about childhood policies at a global scale, despite some strong attempts to catalogue them.^12,13^

 In the face of these challenges, the global research community is making sense of mandatory vaccination in the wake of the pandemic in the same fashion as one eats an elephant: one bite at a time.

## Recent and Current Vaccine Mandate Research

 We are leading work that attempts to tackle some of the most important outstanding questions about mandatory vaccinations. As the pandemic was concluding, one of us (Katie) assembled a team of investigators, designing and securing funding for a multi-million-dollar interdisciplinary evaluation of COVID-19 vaccine mandates. It was no coincidence that this project focuses on the study sites of our earlier article: Australia, Italy, France and California. All adopted significant mandatory policies for COVID-19, as Guaraldi and colleagues^14^ note in the case of Italy. The fact that these jurisdictions layered COVID-19 vaccine mandates over recent new mandatory childhood vaccination policies allowed us to explore the relationship between the two strategies. However, a different selection of cases (including jurisdictions without mandates for childhood, COVID-19 or both) would help to answer other important questions about mandates and their effects.

 Katie also initiated a body of collaborative work to study how similar policies were introduced and implemented in Southeast Asia, which responds to the call by MacDonald et al.^15^ Perhaps the most interesting feature of the mandatory vaccination policies employed in that region is that several governments officially proclaimed that vaccination was voluntary yet introduced numerous limitations on what unvaccinated people could do in terms of work, travel and economic life.^16,17^ Meanwhile, separate work undertaken by Guan^18^ on China illuminates an additional dimension unexplored in Western contexts: what she calls coercive mobilisation, whereby more localised non-state or quasi-state actors pressure individuals to accept vaccines, alongside or instead of top-down government requirements.

 Returning to the COVID-19 vaccine mandate research in our original study jurisdictions, we are attempting to understand the relationships between childhood and COVID-19 vaccine mandates from the perspectives of policy-makers and untangling the various drivers of why specific vaccine mandate policies were introduced and removed at particular times. We are also exploring which contextual factors influenced decisions and examining the crucial question of what mandates achieved in terms of vaccine uptake.

 As we suggest above, the interrelation of preventative policies in a given country’s policy mix is particularly important here. This includes both policies that drive vaccine uptake (additionally and complementary to mandates) and policies that seek to reduce or prevent disease transmission via other methods (such as lockdowns). To this end, some of our recent work has sought to reconceptualise vaccine mandates as having a function beyond coercion and punishment. Analysis of cases in both industrialised and developing countries uncovered that policy-makers frequently used COVID-19 vaccine mandates to reopen economies safely, selectively restoring freedoms and opportunities to the vaccinated that were otherwise denied to everybody.^19^ This is a qualitatively different scenario to the more punitive experience we explored in our original article, where unvaccinated children were allowed to attend educational institutions one day and then denied the next. In a similar vein, Avigur-Eshel describes the operation of vaccine mandates via vaccine certification programs for participation in social and economic activities as an exercise of “promissory legitimacy,” wherein governments make promises about how individuals’ lives will be enhanced and improved tangibly in the future.^5^

 Compared to our prior research, drivers and experiences of mandate removal have become another key focus of COVID-19 vaccine mandate research. Policy termination is generally a rare activity for childhood vaccine mandates (for an exception, see Moran et al^20^) but this is different for emergency policies. Our unpublished early findings indicate numerous and distinctive reasons why COVID-19 vaccine mandates ended when they did, ranging from epidemiological factors to ideology, the public mood, and party-political drivers; some of these factors are also captured in the scholarship on vaccine passports.

 In our own research and beyond, the world these policies leave in their wake is also under interrogation in terms of public opinion about future mandates,^21^ especially at the level of specific groups. Our prior analysis of contestation over California’s childhood mandates found that these policies hardened the views and expectations of both vaccine acceptors and refusers, ultimately shaping future policy contestation.^22^ What will this tendency look like in the context of global pandemic policies? It is also important to study lessons drawn by policy-makers, and court rulings about the legality of mandate instruments and decision-making processes.^23^

## Contested Questions

 Beyond the specific experience of the pandemic, the world-building properties of mandates remain open to contestation, debate, and scholarly interrogation. One open question is how policy takers (those targeted by the policies) experience and internalise messaging or values associated with mandatory vaccination. In discussing public space mandates (“vaccine passports” or requirements for people to be vaccinated to access certain spaces or benefits), Odone and colleagues suggest that this form of governance misses the opportunity to educate the public about why vaccination is good for protecting individual or collective health. Instead, they claim, public space mandates function merely as a mechanistic device to “nudge” individuals who want to access other goods that they see as valuable (ie, social life and entertainment).^3^

 We contend that public space mandates do more than “nudge” members of the public to vaccinate. The essence of a nudge is that one can opt out of a desired behaviour, albeit by exerting some additional effort.^24^ If a negative COVID-19 test can stand in for proof of vaccination, then this fits the definition. However, governments designing public space mandates in Australia, Europe and Asia frequently eschewed or abandoned such opt outs. Vaccine refusers had to forego activities altogether, suggesting that the policies were more coercive than a nudge.

 We also believe that interpreting these policies in terms of their mechanistic operation misses the full picture. Mandates are inevitably accompanied by messaging from government and public health leaders about their aims and effects, such as protecting people (especially the vulnerable), making spaces safe, and allowing venues to open or keep functioning so that businesses and punters benefit. End users therefore encounter coercive policies within a broader discourse about public safety, the interpretation of which is in turn shaped by the lived experiences and perspectives of enforcers and other members of the public. Of course, responses to mandates will vary among individuals and communities. However, our point is that we should not see mandates as necessarily closing off opportunities transmit the benefits of vaccination in concrete and specific ways that connect to the spaces being governed.

## Suggestions for Future Research

 There is so much more work to be done. We would like to see a large-scale analysis of which countries had childhood vaccine mandates and which countries had COVID-19 vaccine mandates, to explore the apparent role of the one influencing the other. We would also like to see research on the ‘bounce-back’ trajectories of countries with and without mandates for childhood vaccines, in terms of vaccine coverage. We also see further need to explore how cultural understandings of voluntarism and compulsion differ in the global south and how this informs vaccine policies and their effectiveness. We would also like to see more analysis of the phenomenon of “coercive mobilisation” identified by Guan^18^ in Western settings. There is also a need for continued attention to the impacts of rhetoric against vaccine mandates, as mobilised by populist disruptors (eg, Robert F. Kennedy Junior). This wishlist demonstrates the ongoing need for political science inputs on study of vaccines, in addition to the other social sciences.

 Perhaps the most important work will consider how to soften the potentially negative impacts and risks of mandates (which are now at least somewhat clearer). Governments will undoubtedly use these instruments again, regardless of what researchers or publics think of the merits!

## Acknowledgements

 The authors thank Alessia Dipalma for her support with finalising this commentary for submission. Katie Attwell acknowledges the wider MandEval team’s ongoing work in the vaccine mandate space.

## Disclosure of artificial intelligence (AI) use

 Not applicable.

## Ethical issues

 Not applicable.

## Conflicts of interest

 Authors declare that they have no conflicts of interest.
